# Transcriptome analysis revealed key genes and pathways related to cadmium tolerance and accumulation in coix (*Coix lacryma-jobi* L.)

**DOI:** 10.3389/fpls.2025.1660959

**Published:** 2026-02-27

**Authors:** Pengliang He, Yucheng Jie, Yi Zeng, Adnan Rasheed, Ningjing Zhu, Zhiyu Xie, Hucheng Xing, Hongdong Jie

**Affiliations:** 1 Institute of Ramie, Hunan Agricultural University, Changsha, Hunan, China; 2 College of Agronomy, Hunan Agricultural University, Changsha, Hunan, China; 3 Hunan Provincial Research Center of Engineering Technology for Grass Crop Germplasm Innovation and Utilization, Changsha, Hunan, China; 4 Hunan Crop Research Institute, Hunan Academy of Agricultural Sciences, Changsha, Hunan, China; 5 Yuelushan Laboratory, Changsha, Hunan, China

**Keywords:** coix, cadmium, stress, transcriptome, gene

## Abstract

Coix (*Coix lacryma-jobi* L.) is an essential medicinal and edible plant with great economic value. Nevertheless, little is known about the molecular mechanisms underlying coix response to cadmium (Cd) stress. Coix germplasm, YY03-03, was exposed to 0 (control), 15, and 30 mg kg^−1^ Cd. We observed that YY03–03 exhibited low Cd absorption and transportation capacity. Further, various enrichment and translocation factors decreased under Cd stress. Moreover, under Cd stress levels of 15 and 30 mg kg^-^¹, transpiration rate, stomatal conductance, and photosynthetic rate significantly decreased (by 54.0% and 64.8%, 40.4% and 47.9%, 27.7% and 37.6%, respectively), while intercellular carbon dioxide concentration significantly increased (by 82.1% and 111.5%, respectively).Next, we conducted a transcriptome analysis of plants in control or 30 mg kg^−1^ Cd group. Transcriptome sequencing generated a total of 6.28–8.79, 7.93–9.37, 8.51–9.79, 6.62–7.38, 7.75–8.47, 7.31–8.37, 6.92–7.75, and 6.40–8.51 billion base pairs (bp) in the control roots, stems, leaves, and grains and Cd-treated roots, stems, leaves, and grains, respectively. Furthermore, 1144, 2924, 3818, and 1702 DEGs were identified in Cd-treated root, stem, leaf, and grain with 682, 942, 1907, and 877 upregulated and 462, 1982, 1911, and 825 downregulated genes, respectively. Quantitative real time-polymerase chain reaction was used to assess 12 stress-responsive differentially expressed genes (DEGs) and validate transcriptomic data. Gene ontology analyses demonstrated that DEGs were primarily engaged in catalytic activity, cellular processes, cell, cell component, binding, and metabolic processes. The Kyoto Encyclopedia of Genes and Genomes pathway analysis revealed that Cd stress altered DEGs primarily involved in environmental adaptability, transport and catabolism, signal transduction, translation, and glucose metabolism. These findings provide a molecular basis for breeding low-Cd coix varieties, which is of significant importance for ensuring the safety of coix as a medicinal and edible resource in Cd-contaminated areas.

## 1 Introduction

Coix belongs to the Poaceae (Gramineae) family and is native to Southeast Asia (Lim, 2012). It is a C4 herb widely grown in China, Japan, India, Thailand, Vietnam, Malaysia, and other countries (Zhang et al., 2023). Coix seeds are rich in amino acids, including the essential amino acids lysine and methionine, which are often deficient in other cereal grains (Lin et al., 2009). The seeds contain a high proportion of non-saturated fatty acids, particularly oleic acid and linoleic acid, which together can constitute up to 75% of the total fatty acid content (Ding et al., 2021). Coix seeds have been utilized in China for approximately 8,000 years, serving both as a food source and for beer brewing (Yang and Jiang, 2010; Wang et al., 2016a). The byproducts of coix processing—namely the bran, bran layer, and husk—serve as valuable oil sources and exhibit significant economic potential and promising development prospects (Feng et al., 2020).

Most importantly, as a plant with medicinal, edible, and fodder uses, coix is renowned as the “King of Gramineous Plants” and the “Pearl of Medicine” due to its exceptionally high medicinal and nutritional value (Devaraj et al., 2020). The grains, roots, and leaves of coix possess medicinal properties and are valued for their roles in immune regulation, antiviral activity, blood pressure reduction, blood glucose management, and antitumor effects (Jinnouchi et al., 2021; Suzuki and Konaya, 2021). As a traditional medicinal agent, coix has been demonstrated to possess significant diuretic and anti-inflammatory properties (Zhu, 2017). Coix seeds have been reported to demonstrate anti-allergic (Hsu et al., 2003; Chen et al., 2010, 2012), anti-obesity (Kim et al., 2004; Huang et al., 2005; Kim et al., 2007; Ha et al., 2010), and antioxidant (Kuo et al., 2001; Yu et al., 2011; Wang et al., 2013, 2016b) properties. Moreover, the total flavonoid content in coix seeds has been reported to be higher than that in other parts of the plant (Huang et al., 2019a). With the renewed recognition of coix’s nutritional and medicinal value, its demand has increased rapidly. Consequently, it has been introduced to nearly all tropical and subtropical regions worldwide (Lim, 2012; Guo et al., 2020).

Given its growing economic and medicinal importance, coix has attracted increasing attention. Currently, China is the world’s largest producer and exporter of coix seed (Huang et al., 2019a). The cultivated area of coix in China currently spans approximately 73,000 hectares, yielding an annual production of around 220,000 tons of grain (Diao, 2017). Currently, coix is primarily cultivated in southern China, with major production areas including Hunan, Hubei, Yunnan, Guizhou, Guangdong, and Guangxi (Huang et al., 2020). Given the high value of coix, the yield and quality safety of its products are of paramount importance. However, soil Cd contamination in major coix production areas (such as Hunan Province) is relatively severe, which has led to a significant decline in coix yield. More importantly, it poses a serious threat to the safety of coix for both food and medicinal purposes, thereby endangering human health. Cd is a non-essential element that impacts the intake and transfer of essential elements, thus inhibiting plant growth by interfering with the absorption and transportation of essential elements (Ma et al., 2021). Cd poses a serious threat to human health as it responsible for various diseases, such as cancer and dysfunction of the kidneys and lungs (Khan et al., 2016). The increasing Cd pollution has led to a substantial Cd accumulation in the food chain (Schreck et al., 2012; Su et al., 2021). To address soil Cd contamination, governments and enterprises have implemented numerous remediation measures, such as phytoremediation technologies. However, these approaches are often associated with certain limitations, including high costs, adverse impacts on soil health, and extended timeframes for effective restoration.

The screening and breeding of crop varieties with low Cd uptake is a low-cost strategy for limiting Cd uptake by humans from contaminated soils (Zhou et al., 2019). Therefore, to mitigate the threat of soil Cd contamination to the safety of coix as both food and medicine and to safeguard human health, it is crucial to develop low-Cd-accumulating coix varieties. Achieving this goal requires a thorough understanding of the mechanisms governing Cd uptake and accumulation in coix. However, studies on the response of coix to Cd stress remain unreported, and the potential mechanisms, particularly the molecular mechanisms, underlying its reaction to Cd stress are still unknown.

High-throughput RNA sequencing (RNA-Seq) offers a powerful approach for capturing genome-wide transcriptional profiles and enables large-scale identification of differentially expressed genes. This technique greatly facilitates the exploration of molecular mechanisms underlying the responses of organisms to biotic or abiotic stresses (Chen et al., 2020). Transcriptome sequencing has been widely employed to investigate plant responses to Cd stress and to elucidate the underlying molecular mechanisms. Transcriptome analysis of wild paper mulberry revealed a significant upregulation of numerous genes under Cd stress. These included key transcription factors such as bHLH and MYB, as well as genes encoding proteins involved in phenylpropanoid biosynthesis and flavonoid biosynthesis, among other pathways (Xu et al., 2019). Transcriptomic analysis of radish roots revealed 1496 differentially expressed genes (DEGs) under Cd stress. These DEGs were primarily engaged in methionine, cysteine, and glucosinolate biosynthesis-related pathways (Xu et al., 2015). Transcriptome analysis revealed key genes and pathways related to Cd-stress tolerance in kenaf (Chen et al., 2020). Therefore, the present study investigated the phenotypic and physiological responses of coix to Cd stress as well as Cd absorption and transportation from root to stem, leaf, and grain. The molecular mechanisms underlying the response of coix to Cd stress were elucidated via transcriptome analysis. Our findings implicated several DEGs and pathways that play vital roles in the Cd stress response of coix.

## 2 Materials and methods

### 2.1 Plant materials and treatment

**Figure 1:**
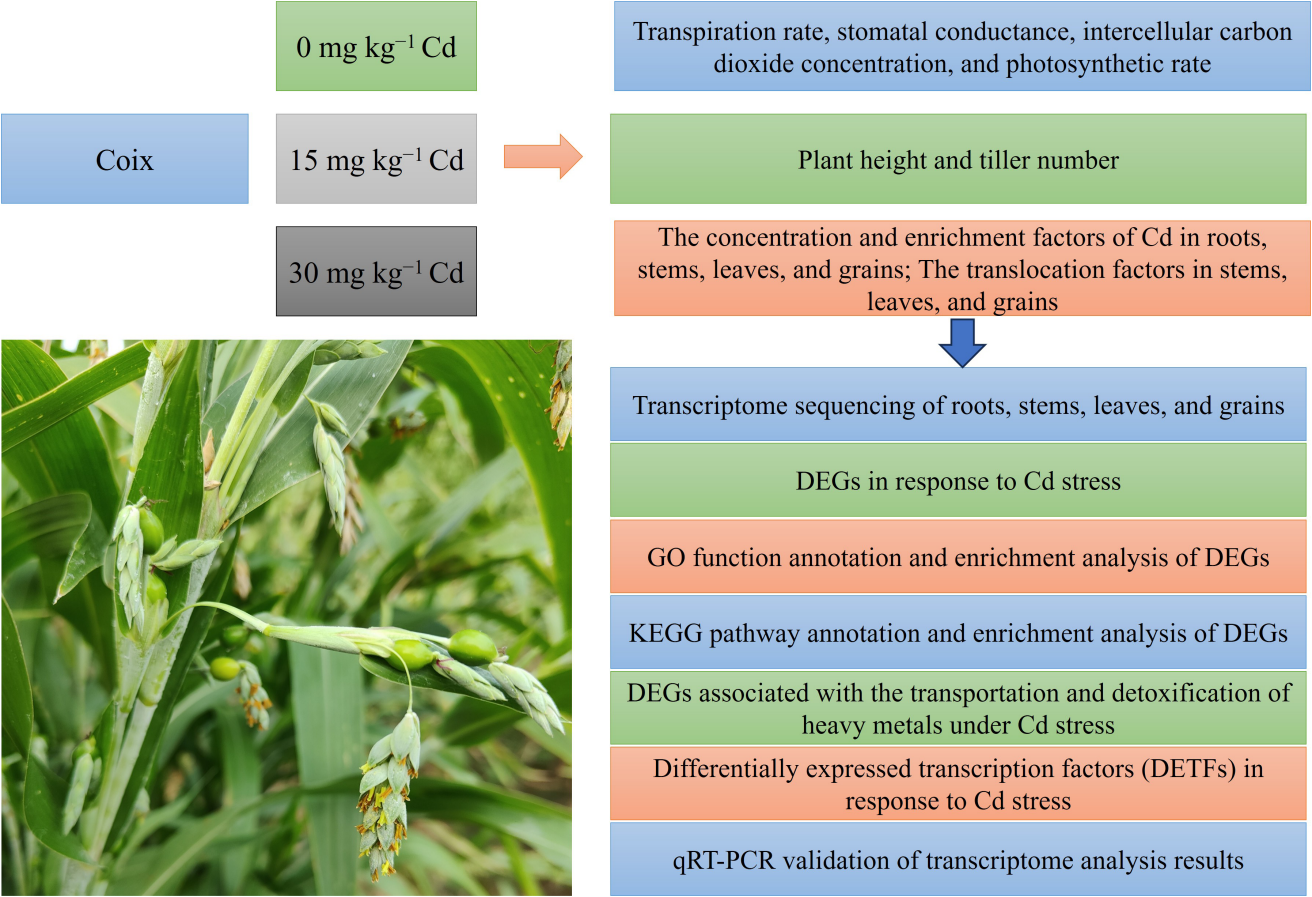
Schematic overview of the experimental design for investigating the response of coix to Cd stress.

A schematic diagram illustrating the overall experimental design is shown in **Figure 1**. We used coix germplasm YY03-03 provided by the Hunan Provincial Research Center of Engineering Technology for Grass Crop Germplasm Innovation and Utilization. Our research group conducted extensive preliminary evaluations of coix germplasm resources, which revealed that the YY03–03 germplasm exhibits three key advantageous characteristics: high yield, strong cadmium tolerance, and low cadmium accumulation capacity. These findings formed the basis for its selection in this study. The soil was taken from the rice experimental field (0∼20 cm) of Hunan Agricultural University. The basic soil characteristics were determined by conventional methods after soil drying (**Table 1**). The soil sample with no added Cd was used as control (CK). The remaining soil samples were treated with either 15 or 30 mg kg^−1^ Cd and expressed as T1 and T2, respectively. Briefly, CdCl_2_·2.5H_2_O was ground in a mortar, and its solution was prepared with a 1/10000 dilution. The solution was loaded into a spray pot and mixed with crushed soil by spraying and mixing in plastic pots (diameter × height = 50 cm × 35 cm, leaky bottom belt tray). A total of 25 kg of soil was filled in each basin, and 50 g of compound fertilizer (NPK; 15% each of nitrogen, phosphorus, and potassium) was applied to each basin. Three biological repeats were used for each treatment, amounting to three plants per pot, nine plants per biological repeat, and 27 plants per treatment. Healthy seeds with full grains and uniform size were disinfected with 0.1 mol/L nitric acid solution for 24 h. The seeds were sown in a nutrition bowl and raised into seedlings till the six-leaf stage. Seedlings with uniform growth were shifted into pots with three plants per pot. The plants were grown under natural light, and conventional water and fertilizer management practices were employed.

**Table 1:** Basic soil characteristics.

Organic matter	Total N	Available N	Total K	Available K	Total P	Available P	pH value	Total Cd
(g kg^−1^)	(g kg^−1^)	(mg kg^−1^)	(g kg^−1^)	(mg kg^−1^)	(g kg^−1^)	(mg kg^−1^)		(mg kg^−1^)
20.24	1.52	225.35	13.49	354.00	1.27	77.07	5.74	0.42

### 2.2 Determination of photosynthetic index

During the filling stage, transpiration rate (Tr), stomatal conductance (GS), intercellular carbon dioxide concentration (Ci), and photosynthetic rate (Pn) of the coix plants were measured using a photosynthesis meter (Li6400XT). The second fully expanded leaf of the main stem from the top was analyzed, and the analyses were done between 9:00 am h and 11:00 am h on a sunny day.

### 2.3 Determination of morphological index

After 111 days of Cd stress treatment, plant height, tiller number, and leaf color, morphology, and growth were measured under varying Cd stresses.

### 2.4 Determination of Cd concentration, enrichment factor, and translocation factor

Dry root, stem, leaf, and grain samples (about 0.5 g per replicate) were crushed and digested separately in 5 mL of a digestive solution containing HNO_3_ and HClO_4_ in a 4:1 ratio. Following digestion, the samples were diluted to 25 mL of deionized H_2_O. Cd concentration was measured using a flame atomic absorption spectrophotometer (ZEEnit 700, Analytik, Germany). We measured Cd (root), Cd (soil), Cd (stem), Cd (leaf), and Cd (grain), which refer to the Cd concentrations in root, soil, stem, leaf, and grain, respectively. Next, we calculated root [REF; Cd (root)/Cd (soil)], stem [SEF; Cd (stem)/Cd (soil)], leaf [LEF; Cd (leaf)/Cd (soil)], and grain enrichment factor [GEF; Cd (grain)/Cd (soil)], stem [STF; Cd (stem)/Cd (root)], leaf [LTF; Cd (leaf)/Cd (root)], and grain translocation factor [GTF; Cd (grain)/Cd (root)].

### 2.5 RNA isolation

Total RNA was extracted from 100 mg coix root, stem, leaf and grain samples using the Trizol reagent kit (Invitrogen, Carlsbad, CA, USA) following the manufacturer’s protocol. The extracted RNA samples were stored at -80°C for subsequent steps such as library construction and sequencing.

### 2.6 Library construction and sequencing

The extracted mRNA (500 ng) was enriched using mRNA Capture Beads. After purification with beads, the mRNA was fragmented using high temperatures. The fragmented mRNA was then used as a template to synthesize the first strand of cDNA in a reverse transcription enzyme mixture system. While synthesizing the second strand of cDNA, end repair and A-tailing were completed. Next, adapters were ligated, and Hieff NGS. DNA Selection Beads were used for purification to select target fragments. PCR library amplification was then performed, and finally, detection was carried out using the Illumina NovaSeq 6000 platform at Gene Denovo Biotechnology Co. (Guangzhou, China).

### 2.7 Preprocessing of sequencing data

Reads obtained from the sequencing machines included raw reads containing adapters or low-quality bases which would affect the following assembly and analysis. Thus, to get high quality clean reads, reads were further filtered by fastp (version 0.18.0) with the following parameters:

1) removing reads containing adapters;
2) removing reads containing more than 10% of unknown nucleotides (N);
3) removing low quality reads containing more than 50% of low quality (Q-value ≤ 20) bases.

The cleaning reads were then assembled *de novo* using the Trinity software (Grabherr et al., 2011) with the parameter –KMER_SIZE 31 –min_kmer_cov 12 –normalize_reads –normalize_max_read_cov 50.

For each transcription region, a FPKM (fragment per kilobase of transcript per million mapped reads) value was calculated to quantify its expression abundance and variations using RSEM software.

### 2.8 Analysis of DEGs

The DEGs in the Cd-treated samples were compared with those in the control samples using the DESeq2 software with significance criteria set as *p*-value < 0.05 and |log_2_FC| ≥ 1.

### 2.9 Functional annotation and analysis

Functions of identified genes were annotated using BlastX with an E value of less than 10^−5^ against gene ontology (GO), Swiss Prot, Kyoto Encyclopedia of Genes and Genomes (KEGG), Cluster of Orthologous Groups (COG), and NCBI non-redundant (Nr) protein databases. GO and KEGG pathway enrichment analyses were performed using Blast2GO and KOBAS2.0, respectively (Kanehisa and Goto, 2000; Conesa et al., 2005).

### 2.10 Quantitative real time-polymerase chain reaction

The expression of 12 Cd stress-related DEGs identified by transcriptomics was confirmed by qRT-PCR. Gene expression profiles were determined in triple biological replicates using the actin gene as the internal control (Wei et al., 2019). The sequences of the primers used for qRT-PCR are listed in **Supplementary Table S1**.

## 3 Results

### 3.1 Changes in coix photosynthesis in response to Cd stress

**Figure 2:**
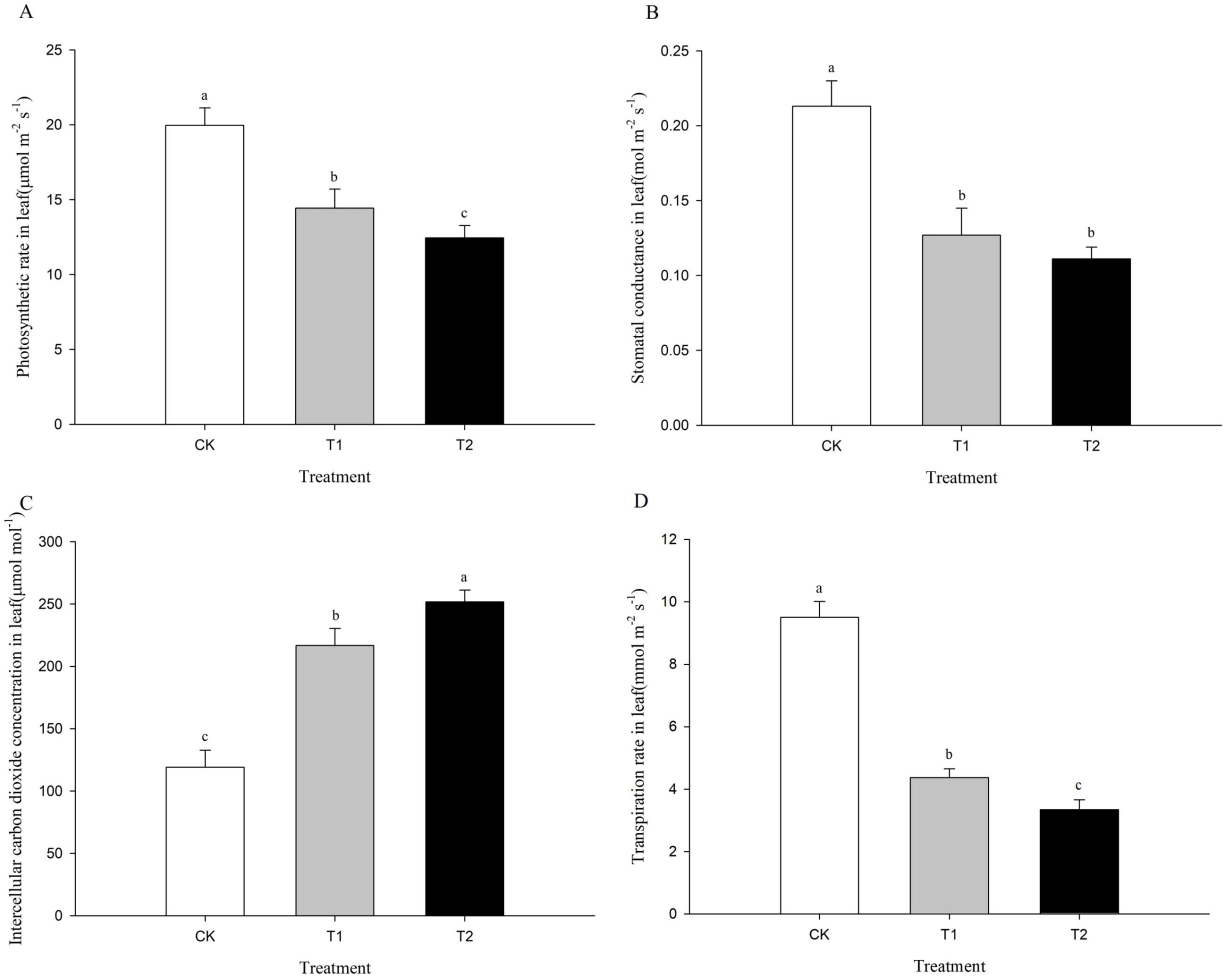
Effects of different Cd concentrations on leaf gas exchange characteristics of coix. Values represent the means of three replications (n = 3) ± SD and different letters indicate significant differences obtained after Duncan’s multiple range test (DMRT) with *p* values < 0.05. Labels CK, T1, and T2 in the figure represent treatment groups with 0, 15, and 30 mg kg^1^ CdCl2, respectively. **(A)** represents photosynthetic rate, **(B)** represents stomatal conductance, **(C)** represents intercellular carbon dioxide concentration, and **(D)** represents transpiration rate.

Compared with controls, under Cd stress of 15 and 30 mg kg^-^¹, Pn, GS, and Tr (19.97 μmol m^−2^ s^−1^, 0.21 mol m^−2^ s^−1^, and 9.50 mmol m^−2^ s^−1^, respectively) decreased significantly by 27.7% and 37.6%, 40.4% and 47.9%, and 54.0% and 64.8%, respectively (P < 0.05), while Ci (119.00 μmol mol^−1^) increased significantly by 82.1% and 111.5% (P < 0.05). (**Figure 2**). The photosynthesis of coix was significantly inhibited under 15 and 30 mg kg^-^¹ Cd stress.

### 3.2 Plant growth in response to Cd stress

**Figure 3:**
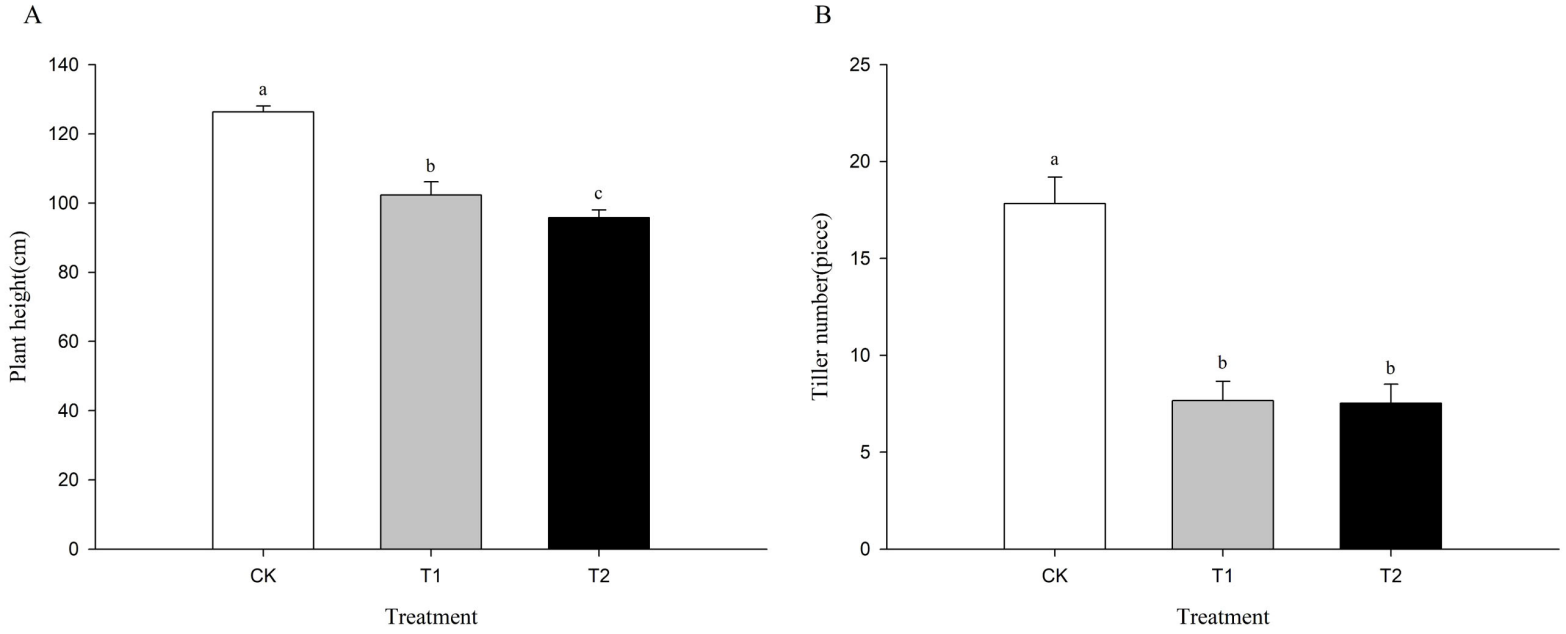
Growth of coix after treatment with 0, 15, and 30 mg kg^−1^ CdCl_2_. Labels CK, T1, and T2 in the figure represent treatment groups with 0, 15, and 30 mg kg^1^ CdCl2, respectively. **(A)** represents plant height, and **(B)** represents tiller number.

Cd-treated plants exhibited significantly reduced growth compared to the control, including reduced plant height and tiller number and yellowish and curled leaves. Compared with controls (with a plant height of 126.37 cm and 17.83 tillers per plant), under Cd stress of 15 and 30 mg kg^-^¹, the plant height and tiller number decreased significantly by factors of 19.0% and 24.2%, and 57.0% and 57.8%, respectively (P < 0.05). The degree of growth inhibition increased with increasing Cd concentration (**Figure 3**). The growth of coix was significantly inhibited under Cd stress at concentrations of 15 and 30 mg kg^-^¹.

### 3.3 Cd absorption and transport in coix

**Figure 4:**
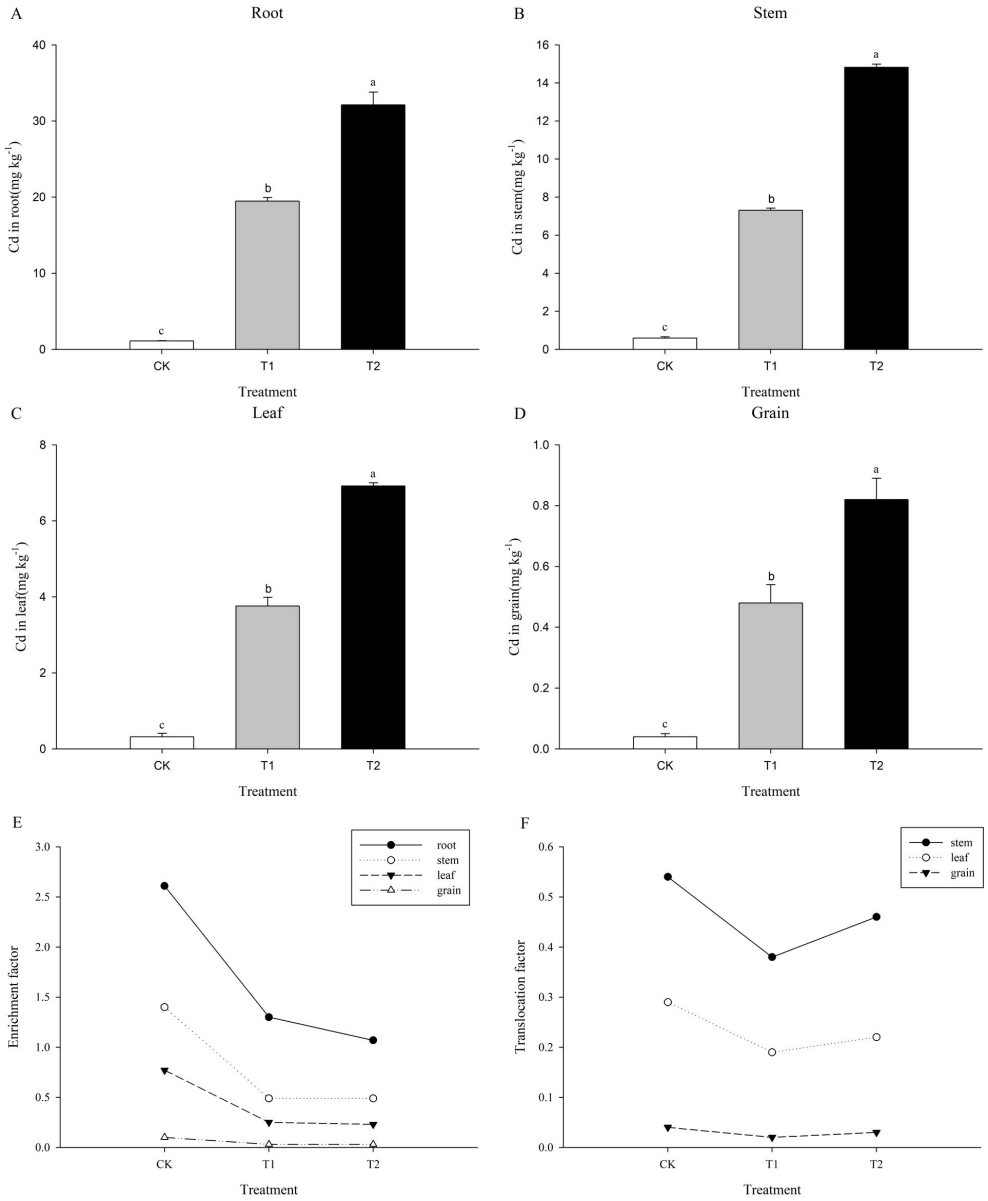
Determination of Cd accumulation. Labels CK, T1, and T2 in the figure represent treatment groups with 0, 15, and 30 mg kg^1^ CdCl2, respectively. **(A)** represents Cd content in the root, **(B)** represents Cd content in the stem, **(C)** represents Cd content in the leaf, **(D)** represents Cd content in the grain, **(E)** represents the Cd enrichment factor, and **(F)** represents the Cd translocation factor.

The concentration of Cd in roots, stems, leaves, and grains was significantly decrease in an order, which was (19.5 and 32.1), (7.3 and 14.8), (3.8 and 6.9), and (0.5 and 0.8) mg kg^−1^ under 15 and 30 mg kg^−1^ Cd, respectively and increased as the Cd stress concentration increased, and the peak of the Cd content was observed under 30 mg kg^−1^ CdCl_2_ treatment (**Figure 4**).

Furthermore, all enrichment and translocation factors decreased with increasing Cd concentration. These findings showed that coix has low Cd absorption and transportation capacity. The Cd enrichment capacity (enrichment factor) was highest in the roots of coix, followed by the stems and leaves, while the grains showed the lowest accumulation ability. The Cd translocation capacity (translocation factor) was highest in the stems of coix, followed by the leaves, while the grains showed the weakest translocation ability.

### 3.4 Transcriptome sequencing

Using the Illumina NovaSeq 6000 platform, a total of 6.28–8.79, 7.93–9.37, 8.51–9.79, 6.62–7.38, 7.75–8.47, 7.31–8.37, 6.92–7.75, and 6.40–8.51 billion bases were acquired from control roots (CK-G), stems (CK-J), leaves (CK-Y), and grains (CK-Z), and Cd-treated roots (C-G), stems (C-J), leaves (C-Y), grains (C-Z), respectively (**Table 2**).

**Table 2:** The throughput and quality of transcriptome sequencing data.

Sample	Raw data (bp)	Clean data (bp)	Q20 (%)	Q30 (%)	GC (%)
CK-1G	8799615000	8682703473	97.49	93.40	56.61
CK-2G	7879046700	7750878741	97.68	93.87	56.49
CK-3G	6283138200	6209319809	98.02	94.37	52.97
C-1G	7752977400	7630090905	97.73	93.94	55.97
C-2G	8198745900	8059707753	97.84	94.11	55.16
C-3G	8478387000	8345707670	97.85	94.20	55.85
CK-1J	9371088300	9279624639	97.90	94.08	53.35
CK-2J	8976588300	8874115057	97.98	94.24	53.24
CK-3J	7933693200	7844425511	98.07	94.46	53.29
C-1J	7852673100	7761337354	97.90	94.11	53.74
C-2J	8372520600	8282070604	97.92	94.13	53.10
C-3J	7311178800	7223285313	97.92	94.16	53.28
CK-1Y	9796827900	9680006685	97.96	94.24	53.82
CK-2Y	9012743700	8908893486	98.01	94.35	53.81
CK-3Y	8511408000	8417851481	98.09	94.52	53.39
C-1Y	7121194200	7032941212	97.96	94.26	54.45
C-2Y	6920555700	6849480736	98.01	94.38	53.88
C-3Y	7759281900	7666414108	97.81	93.89	53.74
CK-1Z	7367529900	7277744782	97.75	93.73	53.71
CK-2Z	6624324300	6544377218	98.00	94.30	53.58
CK-3Z	7382218800	7298322289	97.95	94.23	53.37
C-1Z	6755741400	6674008792	97.93	94.17	53.81
C-2Z	6404180100	6333433032	98.00	94.34	52.54
C-3Z	8510092200	8416428804	97.93	94.13	53.05

After filtration and trimming, 6.20–8.68, 7.63–8.34, 7.84–9.27, 7.22–8.28, 8.41–9.68, 6.84–7.66, 6.54–7.29, and 6.33–8.41 billion bases were acquired from CK-G, C-G, CK-J, C-J, CK-Y, C-Y, CK-Z, and C-Z, respectively. The Q20 and Q30 values were more than 97% and 93%, respectively, demonstrating the high quality of transcriptome sequencing data. The GC contents were about 52.54%–56.61% (**Table 2**).

### 3.5 DEGs in response to Cd stress

**Figure 5:**
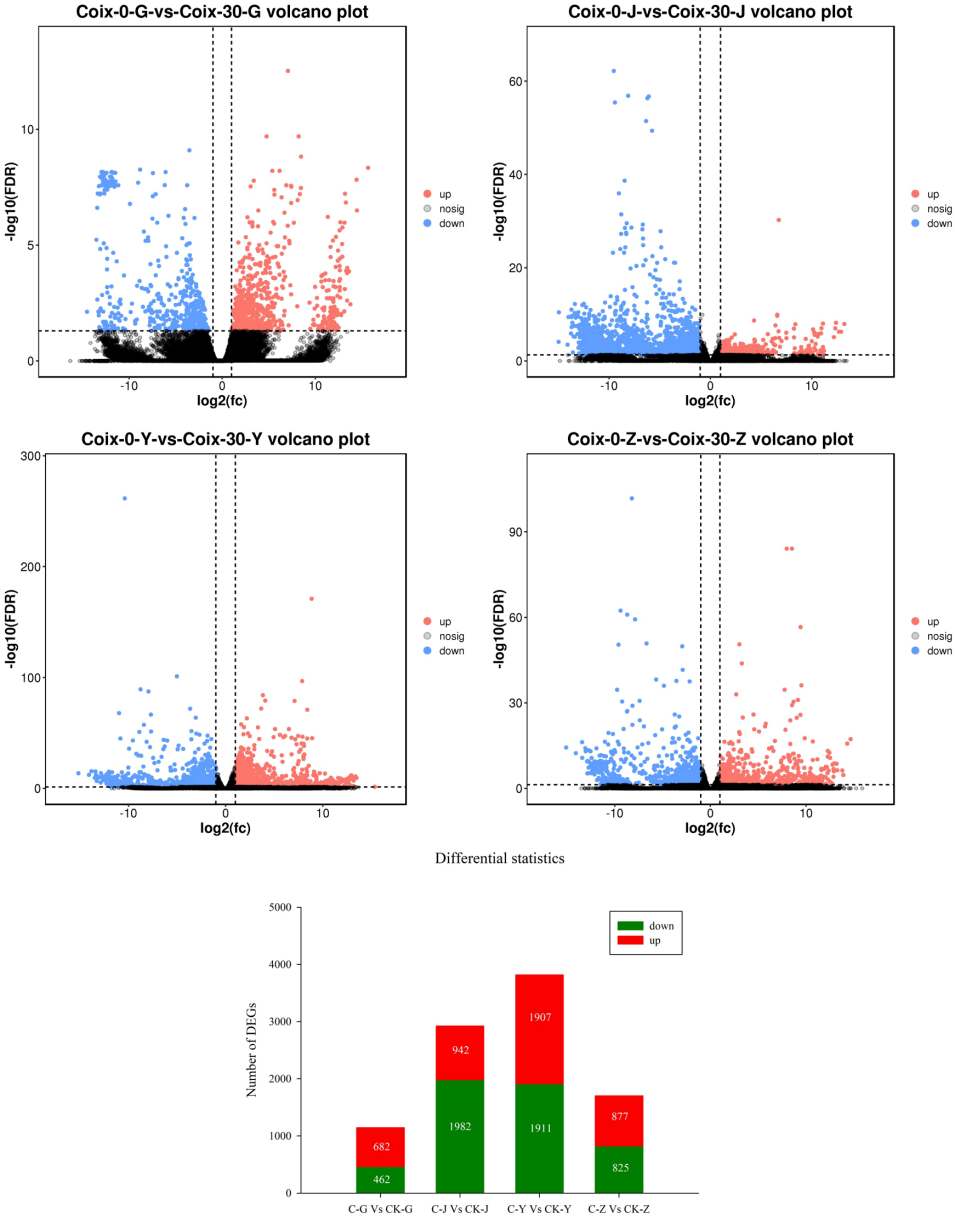
Differentially expressed genes (DEGs) between Cd-treated and control plants. Note: Coix-0-G-vs-Coix-30-G, Coix-0-J-vs-Coix-30-J, Coix-0-Y-vs-Coix-30-Y, Coix-0-Z-vs-Coix-30-Z, where G, J, Y, and Z represent gene expression in the root, stem, leaf, and grain, respectively.

In total, 1144, 2924, 3818, and 1702 genes were differentially expressed in the Cd-treated roots, stems, leaves, and grains compared to controls, with 682 (59.62%), 942 (32.22%), 1907 (49.95%), and 877 (51.53%) upregulated and 462 (40.38%), 1982 (67.78%), 1911 (50.05%), and 825 (48.47%) downregulated genes, respectively (**Figure 5**; **Supplementary Tables S2–S5**).

The number of downregulated genes in the Cd-treated stems was 2.10 times the number of upregulated genes, indicating that most genes were repressed under Cd stress. These findings indicated that Cd stress substantially influenced gene expression.

### 3.6 GO function annotation and enrichment analysis of DEGs

**Figure 6:**
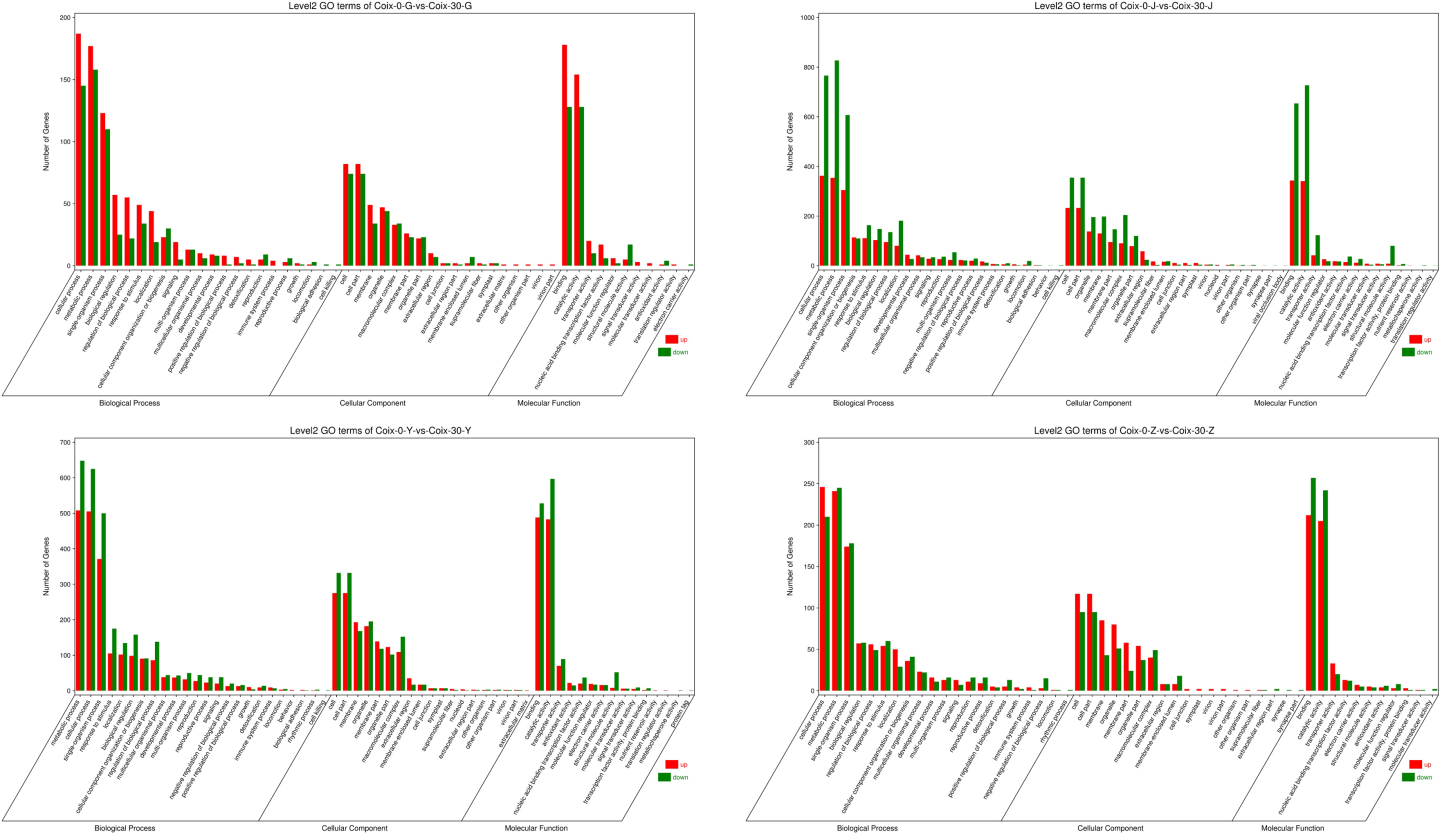
Gene ontology (GO) function annotation analysis of the differentially expressed genes (DEGs). Note: Coix-0-G-vs-Coix-30-G, Coix-0-J-vs-Coix-30-J, Coix-0-Y-vs-Coix-30-Y, Coix-0-Z-vs-Coix-30-Z, where G, J, Y, and Z represent gene expression in the root, stem, leaf, and grain, respectively.

In root, stem, leaf, and grain, the DEGs were categorized into 51, 58, 58 and 51 functional groups, respectively, including “biological process” (BP), “cellular component” (CC), and “molecular function” (MF), with 22, 23, 24, and 21; 18, 21, 19, and 19; and 11, 14, 15, and 11 subcategories, respectively (**Figure 6**; **Supplementary Tables S6–S9**).

In BP, the majority of GO terms were clustered into metabolic process, biological regulation, localization, single organism process, regulation of biological process, cellular process, cellular component organization or biogenesis subcategories, and response to stimulus. In CC, the top seven subcategories were organelle part, membrane part, macromolecular complex, organelle, membrane, cell part, and cell. In MF, the transporter activity, catalytic activity, and binding subcategories were dominant. In particular, “antioxidant activity” in MF and “response to stimulus,” “signaling,” and “detoxification” in BP were functionally annotated with five, 83, 24, and six DEGs in root, respectively. These DEGs play vital roles in plant stress tolerance. A total of 35, 274, 62, and 16; 37, 280, 58, and 23; and ten, 114, 20, and nine DEGs were identified in stems, leaves, and grains, respectively.

**Figure 7:**
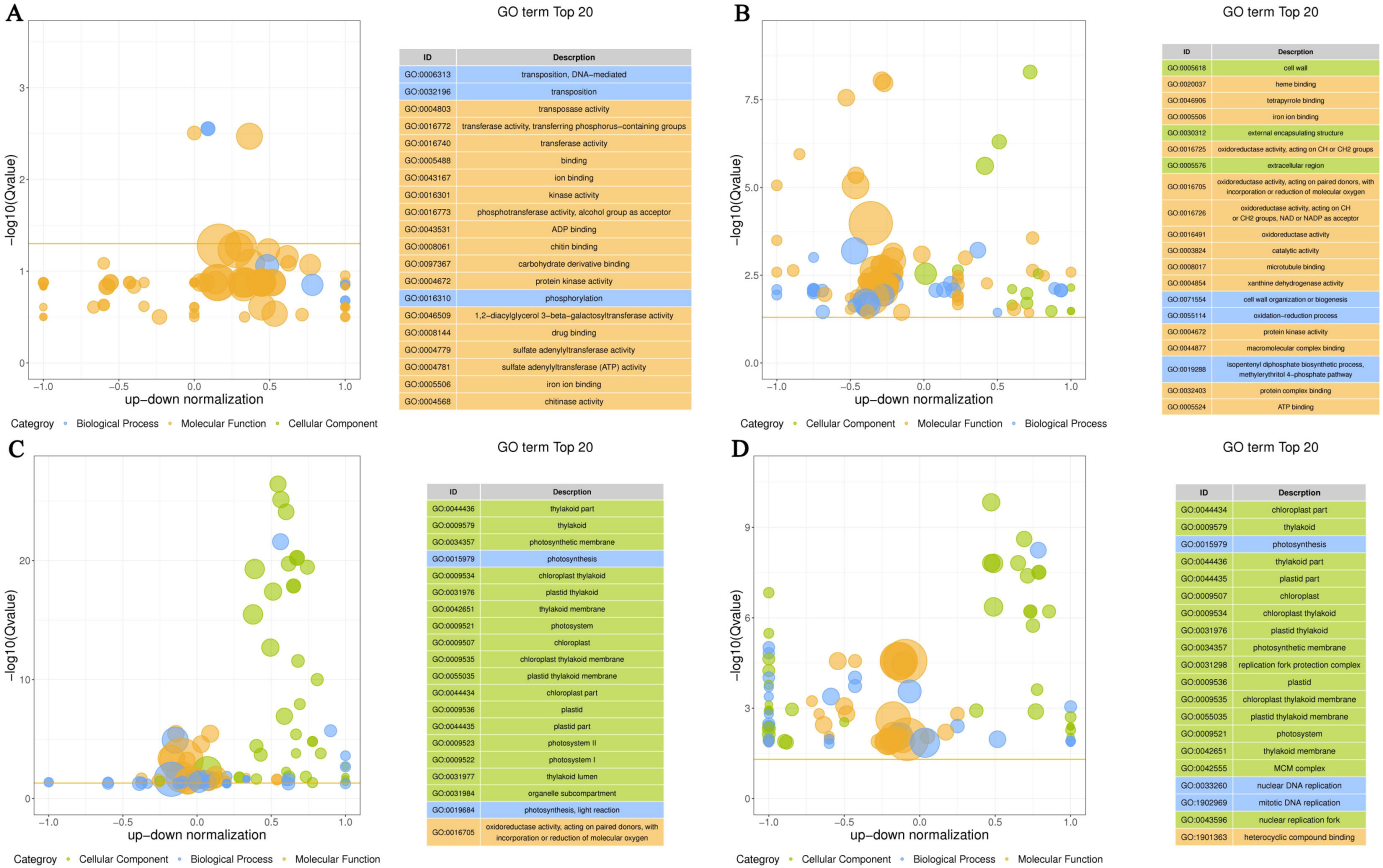
Gene ontology (GO) enrichment analysis of the differentially expressed genes (DEGs). **(A-D)** represent gene expression in the root, stem, leaf, and grain, respectively.

The first four GO terms significantly enriched for DEGs between control and Cd-treated roots were transposition, DNA-mediated (GO: 0006313), transposition (GO: 0032196), transposase activity (GO: 0004803), and transferase activity (GO: 0016740) (**Figure 7**). In stems, the significantly enriched GO terms were cell wall (GO: 0005618), heme binding (GO: 0020037), iron ion binding (GO: 0005506), and tetrapyrrole binding (GO: 0046906). In leaves, the significantly enriched GO terms were thylakoid part (GO: 0044436), thylakoid (GO: 0009579), photosynthetic membrane (GO: 0034357), and photosynthesis (GO: 0015979). In grains, the significantly enriched GO terms were chloroplast part (GO: 0044434), thylakoid (GO: 0009579), photosynthesis (GO: 0015979), and thylakoid part (GO: 0044436). These data showed variable responses of roots, stems, leaves and grains to Cd stress. The responses of both leaves and grains collectively revealed the core harm of Cd stress—a severe inhibition of photosynthesis, thereby directly affecting plant growth.

### 3.7 KEGG pathway annotation and enrichment analysis of DEGs

**Figure 8:**
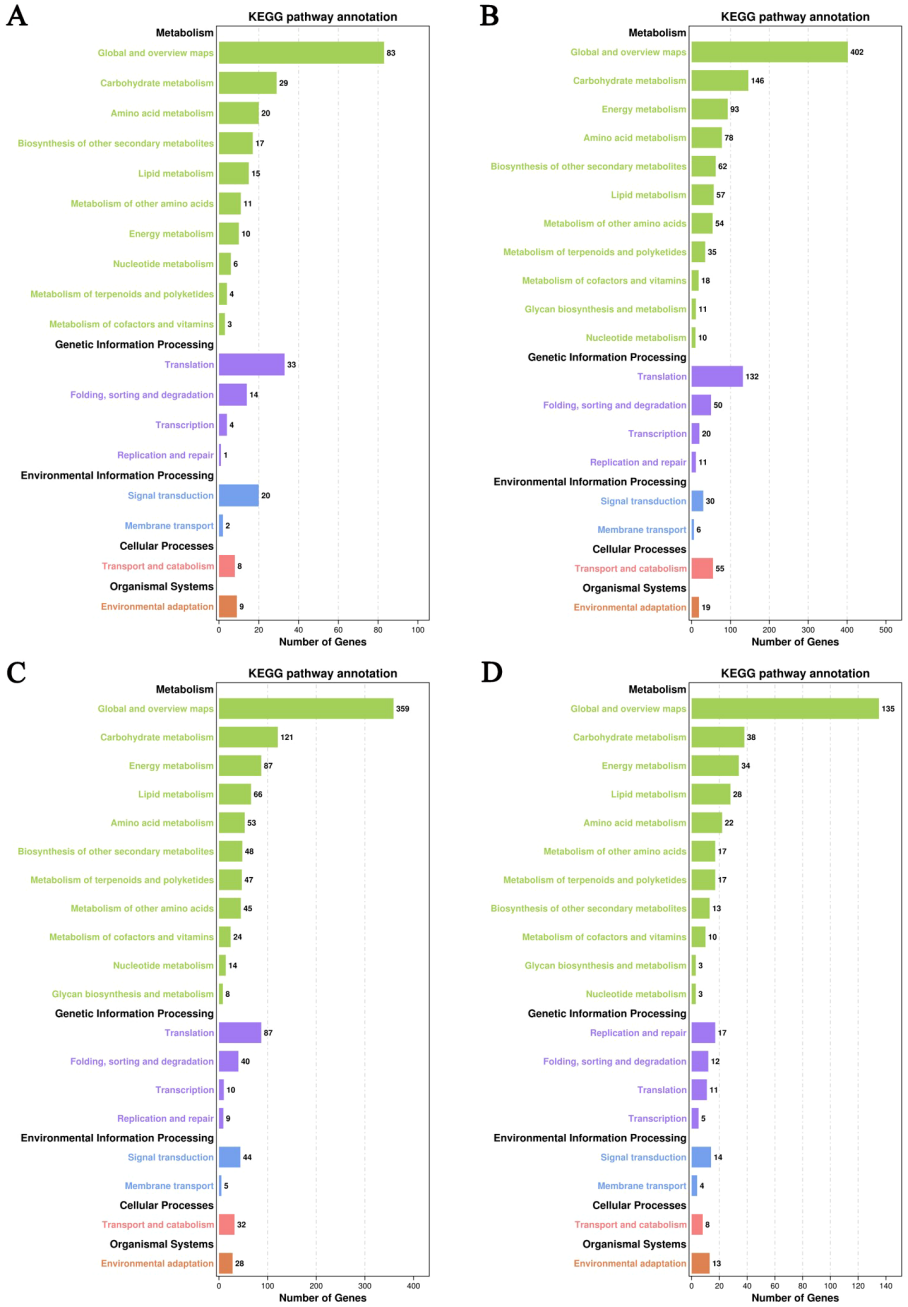
Kyoto Encyclopedia of Genes and Genomes (KEGG) annotation analysis of the differentially expressed genes (DEGs). **(A-D)** represent gene expression in the root, stem, leaf, and grain, respectively.

All DEGs in roots, stems, leaves, and grains were mapped to 87, 120, 116, and 96 KEGG pathways, respectively. The pathways are divided into five categories, which were further divided into 18, 19, 19, and 19 subcategories for roots, stems, leaves, and grains, respectively (**Figure 8**; **Supplementary Tables S10–S13**). Cd stress affected subcategories “carbohydrate metabolism,” “translation” (root, stem, and leaf) or “replication and repair” (grain), “signal transduction,” “transport and catabolism,” and “environmental adaptation.” In addition, the DEGs were concentrated in the “energy metabolism,” “lipid metabolism,” and “amino acids metabolism” subcategories. These subcategories are related to plant responses to plant growth and development and environmental stimuli.

**Figure 9:**
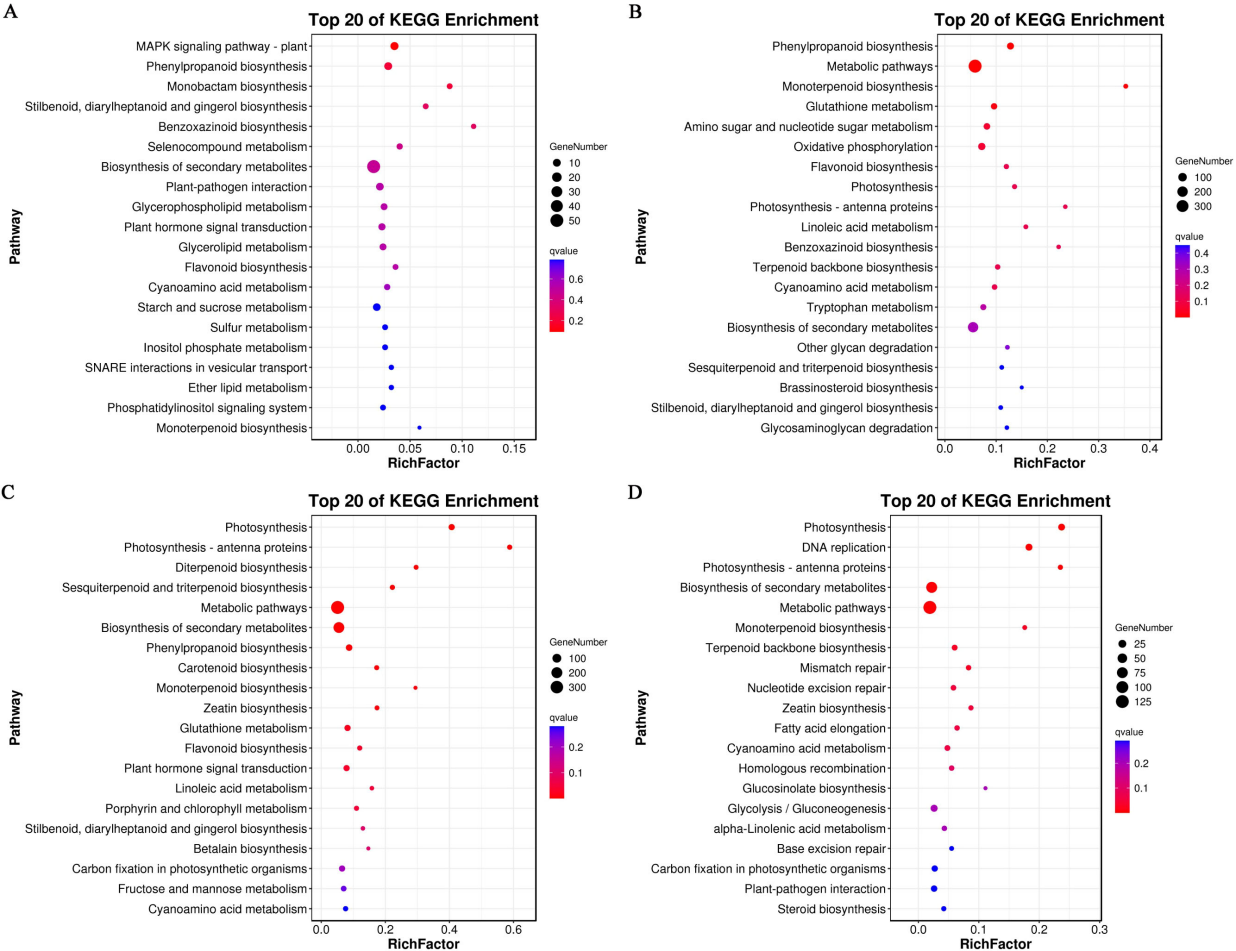
Kyoto Encyclopedia of Genes and Genomes (KEGG) enrichment analysis of the differentially expressed genes (DEGs). **(A-D)** represent gene expression in the root, stem, leaf, and grain, respectively.

In Cd-treated roots, the DEGs primarily enriched in biosynthesis of secondary metabolites (51 DEGs), phenylpropanoid biosynthesis (ten DEGs), MAPK signaling pathway–plant (11 DEGs), plant-pathogen interaction (nine DEGs), plant hormone signal transduction (seven DEGs), flavonoid biosynthesis (three DEGs), and others (**Figure 9**; **Supplementary Table S14**).

In Cd-treated stems, the main altered pathways included biosynthesis of secondary metabolites (192 DEGs), metabolic pathways (387 DEGs), phenylpropanoid biosynthesis (44 DEGs), flavonoid biosynthesis (ten DEGs), glutathione metabolism (28 DEGs), photosynthesis (eight DEGs), photosynthesis-antenna proteins (four DEGs), tryptophan metabolism (22 DEGs), and others (**Figure 9**; **Supplementary Table S15**). A total of 22 DEGs (5 up-regulated and 17 down-regulated) were identified in the tryptophan metabolic pathway. Tryptophan serves as a precursor for the synthesis of key hormones such as auxin (IAA). Auxin is responsible for regulating plant growth, development, and stress responses. Alterations in this pathway indicate that Cd stress interferes with auxin synthesis, thereby inhibiting the growth of coix seeds.

In Cd-treated leaves, the primarily altered pathways included photosynthesis (24 DEGs), photosynthesis-antenna proteins (ten DEGs), phenylpropanoid biosynthesis (30 DEGs), biosynthesis of secondary metabolites (193 DEGs), metabolic pathways (334 DEGs), plant hormone signal transduction (24 DEGs), glutathione metabolism (24 DEGs), flavonoid biosynthesis (ten DEGs), and others (**Figure 9**; **Supplementary Table S16**).

In Cd-treated grains, the main altered pathways included biosynthesis of secondary metabolites (78 DEGs), photosynthesis (14 DEGs), photosynthesis-antenna proteins (four DEGs), plant-pathogen interaction (11 DEGs), metabolic pathways (128 DEGs), and others (**Figure 9**; **Supplementary Table S17**).

### 3.8 DEGs associated with the transportation and detoxification of heavy metals under Cd stress

Under abiotic stress, transporters play vital roles in absorbing and transporting heavy metals and plant metabolism. The present study identified 30 transporter genes, including ABC (ATP-binding cassette transporter), in the root. Among them are four heavy metal transporters (HMTs). We observed a significant upregulation of all these HMTs under Cd stress, including Unigene0015490, Unigene0048533, Unigene0063149, and Unigene0101091 (Log_2_FoldChange = 1.79, 2.18, 1.96, and 2.19, respectively; **Supplementary Tables S18, S19**). Furthermore, six genes were identified to be associated with detoxification, including five upregulated genes and one downregulated gene.

In total, 165 transporter genes, including MTP (metal tolerance protein), ABC, ZIP (zinc and iron regulated transporter proteins), and CAX (cation exchanger), were identified in the stem. This subset included 18 HMTs. Among them, six genes were significantly upregulated under Cd stress, including Unigene0004542, Unigene0063912, Unigene0071261, Unigene0092412, Unigene0095544, and Unigene0095545 (Log_2_FoldChange = 1.25, 1.67, 1.83, 3.35, 1.93, and 1.55, respectively; **Supplementary Tables S20 and S21**). Furthermore, 16 genes were identified to be associated with detoxification, including six upregulated and ten downregulated genes.

Furthermore, 159 transporter genes, such as ABC, ZIP, YSL (yellow stripe-like transporter), and CAX, were identified in the leaf. This subset included 13 HMTs. Among them, seven genes were significantly upregulated under Cd stress, including Unigene0002853, Unigene0044711, Unigene0051971, Unigene0090192, Unigene0092438, Unigene0099561, and Unigene0099564 (Log_2_FoldChange = 1.46, 2.16, 1.31, 1.97, 7.37, 1.42, 2.04, respectively; **Supplementary Tables S22, S23**). Furthermore, 23 genes were identified to be associated with detoxification, including nine upregulated and 14 downregulated genes.

Finally, 53 transporter genes, such as ABC, were identified in the grain. This subset contained nine HMTs. Of them, four genes were significantly upregulated under Cd stress, including Unigene0006961, Unigene0016620, Unigene0051971, Unigene0094764 (Log_2_FoldChange = 2.49, 2.15, 1.37, and 1.34, respectively; **Supplementary Tables S24, S25**). Furthermore, nine genes were identified to be associated with detoxification, including five upregulated and four downregulated genes.

### 3.9 Differentially expressed transcription factors in response to Cd stress

Previous studies reported several DETFs under Cd stress, such as MYB, WRKY, bHLH, NAC, and C2H2 family. In the present study, 1549 TFs from 54 families were identified in Cd-treated samples. Among them, C2H2, bHLH, bZIP, and ERF were identified as the four largest TF families with 153, 123, 119, and 119 TFs, respectively. The HSF, WRKY, GRAS, MYB, and NAC TFs, being responsive to a variety of stresses, were also characterized as large families, with 25, 87, 70, 68 and 90 members, respectively (**Table 3**).

**Table 3:** Differentially expressed transcription factors (DETFs) under cadmium (Cd) stress.

Organ	TF family	Total number of characterized TFs	Total number of differentially expressed TFs	Number of upregulated TFs	Number of downregulated TFs
Root	C2H2	153	2	2	0
	bHLH	123	2	1	1
	bZIP	119	2	0	2
	ERF	119	3	3	0
	MYB_related	90	2	1	1
	NAC	90	1	1	0
	WRKY	87	4	3	1
	GRAS	70	3	2	1
	MYB	68	3	2	1
	FAR1	54	2	1	1
	B3	42	0	0	0
	C3H	40	2	1	1
	G2-like	35	0	0	0
	Trihelix	33	5	3	2
	Dof	29	0	0	0
	HD-ZIP	28	1	1	0
	GATA	27	0	0	0
	HSF	25	2	1	1
	LBD	22	1	1	0
	ARF	22	1	1	0
	TCP	18	0	0	0
	MIKC	17	0	0	0
	SBP	16	1	1	0
	TALE	16	0	0	0
	GeBP	16	1	1	0
	M-type	14	0	0	0
	ZF-HD	13	0	0	0
	NF-YB	13	0	0	0
	NF-YC	12	0	0	0
	HB-other	11	0	0	0
	AP2	10	0	0	0
	NF-YA	10	0	0	0
	YABBY	9	0	0	0
	EIL	9	0	0	0
	CO-like	9	0	0	0
	GRF	8	0	0	0
	Nin-like	7	0	0	0
	DBB	7	0	0	0
	E2F/DP	7	0	0	0
	SRS	6	1	1	0
	LSD	6	0	0	0
	CPP	6	0	0	0
	ARR-B	6	0	0	0
	BES1	4	0	0	0
	CAMTA	4	0	0	0
	WOX	3	0	0	0
	RAV	3	0	0	0
	NF-X1	3	0	0	0
	Whirly	2	0	0	0
	VOZ	2	0	0	0
	HB-PHD	2	0	0	0
	BBR-BPC	2	0	0	0
	S1Fa-like	1	0	0	0
	HRT-like	1	0	0	0
	total	1549	39	27	12
Stem	C2H2	153	14	1	13
	bHLH	123	14	7	7
	bZIP	119	11	3	8
	ERF	119	3	1	2
	MYB_related	90	1	0	1
	NAC	90	6	1	5
	WRKY	87	11	0	11
	GRAS	70	2	1	1
	MYB	68	3	3	0
	FAR1	54	1	0	1
	B3	42	2	2	0
	C3H	40	0	0	0
	G2-like	35	1	1	0
	Trihelix	33	0	0	0
	Dof	29	1	1	0
	HD-ZIP	28	5	4	1
	GATA	27	2	2	0
	HSF	25	1	0	1
	LBD	22	3	2	1
	ARF	22	0	0	0
	TCP	18	1	1	0
	MIKC	17	4	1	3
	SBP	16	0	0	0
	TALE	16	0	0	0
	GeBP	16	0	0	0
	M-type	14	1	0	1
	ZF-HD	13	1	1	0
	NF-YB	13	0	0	0
	NF-YC	12	0	0	0
	HB-other	11	0	0	0
	AP2	10	0	0	0
	NF-YA	10	0	0	0
	YABBY	9	0	0	0
	EIL	9	1	1	0
	CO-like	9	1	1	0
	GRF	8	0	0	0
	Nin-like	7	0	0	0
	DBB	7	0	0	0
	E2F/DP	7	1	1	0
	SRS	6	0	0	0
	LSD	6	1	1	0
	CPP	6	1	1	0
	ARR-B	6	0	0	0
	BES1	4	0	0	0
	CAMTA	4	0	0	0
	WOX	3	1	0	1
	RAV	3	0	0	0
	NF-X1	3	0	0	0
	Whirly	2	0	0	0
	VOZ	2	0	0	0
	HB-PHD	2	0	0	0
	BBR-BPC	2	0	0	0
	S1Fa-like	1	0	0	0
	HRT-like	1	0	0	0
	total	1549	94	37	57
Leaf	C2H2	153	4	2	2
	bHLH	123	10	4	6
	bZIP	119	7	1	6
	ERF	119	8	4	4
	MYB_related	90	6	3	3
	NAC	90	10	4	6
	WRKY	87	11	3	8
	GRAS	70	2	1	1
	MYB	68	7	2	5
	FAR1	54	2	1	1
	B3	42	3	3	0
	C3H	40	0	0	0
	G2-like	35	3	2	1
	Trihelix	33	2	2	0
	Dof	29	3	1	2
	HD-ZIP	28	1	0	1
	GATA	27	3	2	1
	HSF	25	1	0	1
	LBD	22	3	1	2
	ARF	22	3	0	3
	TCP	18	1	0	1
	MIKC	17	1	1	0
	SBP	16	1	0	1
	TALE	16	2	0	2
	GeBP	16	0	0	0
	M-type	14	1	0	1
	ZF-HD	13	1	1	0
	NF-YB	13	0	0	0
	NF-YC	12	0	0	0
	HB-other	11	1	1	0
	AP2	10	1	1	0
	NF-YA	10	0	0	0
	YABBY	9	1	1	0
	EIL	9	0	0	0
	CO-like	9	4	3	1
	GRF	8	1	0	1
	Nin-like	7	0	0	0
	DBB	7	0	0	0
	E2F/DP	7	0	0	0
	SRS	6	0	0	0
	LSD	6	0	0	0
	CPP	6	0	0	0
	ARR-B	6	0	0	0
	BES1	4	0	0	0
	CAMTA	4	0	0	0
	WOX	3	0	0	0
	RAV	3	0	0	0
	NF-X1	3	0	0	0
	Whirly	2	0	0	0
	VOZ	2	0	0	0
	HB-PHD	2	0	0	0
	BBR-BPC	2	0	0	0
	S1Fa-like	1	0	0	0
	HRT-like	1	0	0	0
	total	1549	104	44	60
Grain	C2H2	153	1	0	1
	bHLH	123	5	2	3
	bZIP	119	4	3	1
	ERF	119	3	0	3
	MYB_related	90	1	1	0
	NAC	90	3	1	2
	WRKY	87	6	1	5
	GRAS	70	1	0	1
	MYB	68	2	2	0
	FAR1	54	3	1	2
	B3	42	0	0	0
	C3H	40	0	0	0
	G2-like	35	1	1	0
	Trihelix	33	1	1	0
	Dof	29	1	0	1
	HD-ZIP	28	1	1	0
	GATA	27	1	1	0
	HSF	25	0	0	0
	LBD	22	2	2	0
	ARF	22	1	1	0
	TCP	18	1	1	0
	MIKC	17	0	0	0
	SBP	16	0	0	0
	TALE	16	1	1	0
	GeBP	16	0	0	0
	M-type	14	1	1	0
	ZF-HD	13	1	1	0
	NF-YB	13	0	0	0
	NF-YC	12	0	0	0
	HB-other	11	1	1	0
	AP2	10	0	0	0
	NF-YA	10	0	0	0
	YABBY	9	1	1	0
	EIL	9	0	0	0
	CO-like	9	0	0	0
	GRF	8	0	0	0
	Nin-like	7	0	0	0
	DBB	7	0	0	0
	E2F/DP	7	0	0	0
	SRS	6	0	0	0
	LSD	6	0	0	0
	CPP	6	0	0	0
	ARR-B	6	0	0	0
	BES1	4	0	0	0
	CAMTA	4	0	0	0
	WOX	3	0	0	0
	RAV	3	0	0	0
	NF-X1	3	0	0	0
	Whirly	2	0	0	0
	VOZ	2	0	0	0
	HB-PHD	2	0	0	0
	BBR-BPC	2	0	0	0
	S1Fa-like	1	0	0	0
	HRT-like	1	0	0	0
	total	1549	43	24	19

Under Cd stress, expression analysis revealed that 39 TFs were significantly regulated in root. Among them, ERF, WRKY, GRAS, MYB, and Trihelix were ranked in the top five with three, four, three, three, and five DETFs, respectively. Interestingly, under Cd stress, most genes were found to be upregulated (682 out of 1144, accounting for 59.62%) and fewer genes were downregulated (**Figure 5**). Similarly, for most TF-encoding DEGs (27 out of 39, accounting for 81.69%) were upregulated (**Table 3**).

Under Cd stress, 94 TFs were significantly regulated in the stem. Among them, C2H2, bHLH, bZIP, WRKY, and NAC were ranked in the top five, with 14, 14, 11, 11, and six DETFs, respectively. Interestingly, under Cd stress, most genes were found to be downregulated and fewer genes (942 out of 2924, accounting for 32.22%) were upregulated (**Figure 5**). Similarly, fewer TF-related DEGs (37 out 94, accounting for 39.36%) were upregulated (**Table 3**).

Under Cd stress, 104 TFs were significantly regulated in the leaf. Among them, WRKY, bHLH, NAC, ERF, bZIP, and MYB were ranked in the top six with 11, ten, ten, eight, seven, and seven DETFs, respectively. Under Cd stress, about half of the genes (1907 out of 3818, accounting for 49.95%) were upregulated (**Figure 5**). Similarly, nearly half of the TF-encoding DEGs (44 out of 104, or 42.31%) were upregulated (**Table 3**).

Finally, 43 TFs were significantly regulated in grain. Among them, WRKY, bHLH, bZIP, ERF, NAC, and FAR1 were ranked in the top six with six, five, four, three, three, and three DETFs, respectively. Under Cd stress, around half of the genes (877 out of 1702, accounting for 51.53%) were upregulated (**Figure 5**). Similarly, nearly half of the TF-encoding DEGs (24 out of 43, or 55.81%) were upregulated (**Table 3**).

### 3.10 qRT-PCR validation of transcriptome analysis results

**Figure 10:**
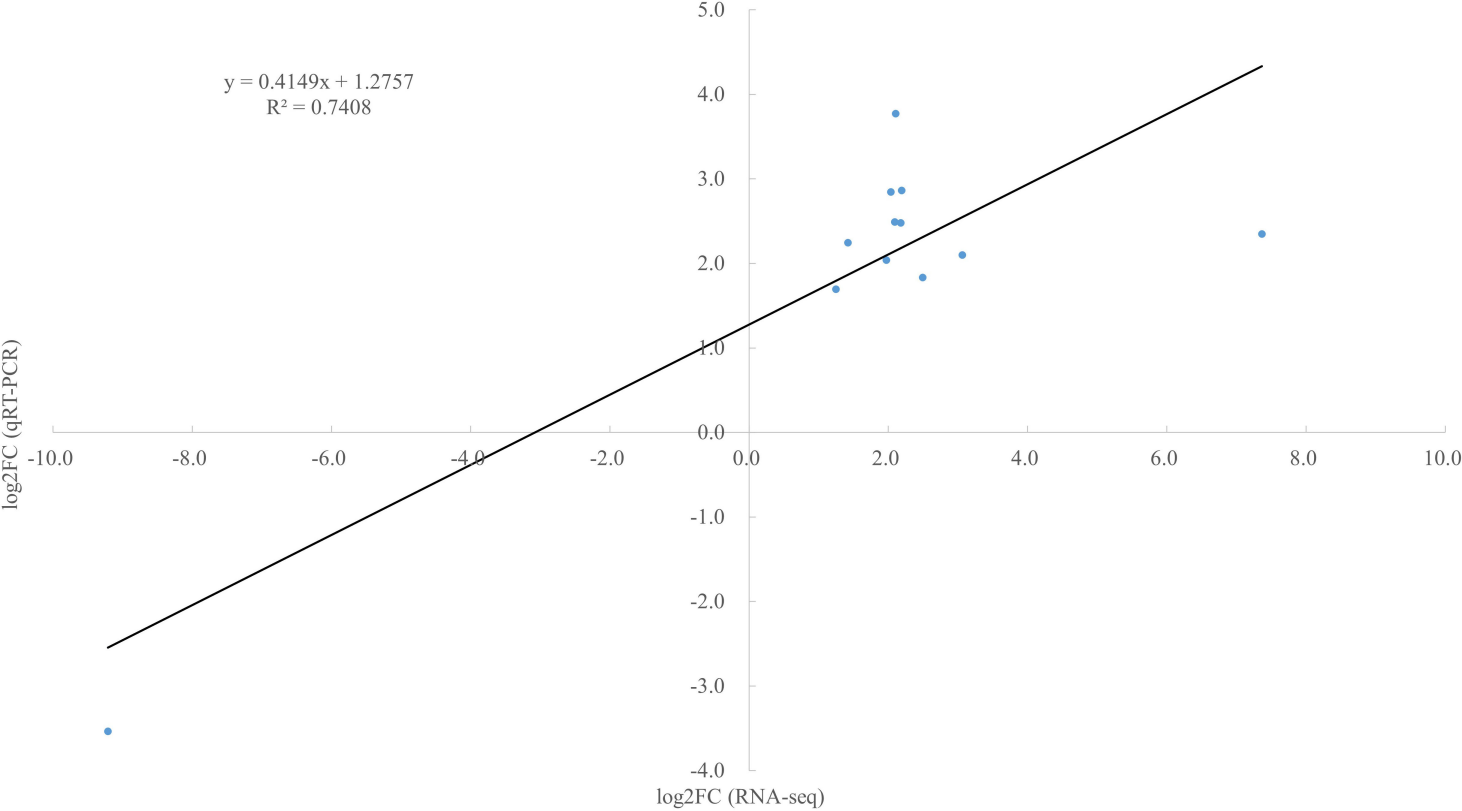
Quantitative real time-polymerase chain reaction (qRT-PCR) validation of 12 differentially expressed unigenes from root, stem, leaf, and grain.

To experimentally validate the reliability of our transcriptome profiling data, we performed qRT-PCR analysis on 12 key DEGs implicated in Cd response and flavonoid biosynthesis. The selection criteria for these genes were based on their significant fold-changes and pivotal roles in the relevant biological pathways. The results demonstrated a strong concordance between the qRT-PCR data and the RNA-seq findings (**Figure 10**). This high degree of consistency confirms the accuracy and reproducibility of our transcriptomic data. Specifically, the validated genes include: Eight transporter-related genes: such as ABCG28 (Unigene0048533) and ABCC3 (Unigene0101091) in roots, MTP4 (Unigene0004542) in stems, and IRT1 (Unigene0090192), ABCA7 (Unigene0092438), YSL13 (Unigene0099561) and YSL12 (Unigene0099564) in leaves, ABCB11 (Unigene0006961) in grains. Four flavonoid biosynthetic genes: including HCT4 (Unigene0093309) in roots and CYP93G1 (Unigene0035743), CYP75B3 (Unigene0056120) and CYP73A12 (Unigene0090121) in stems. The successful validation of these key DEGs reinforces the credibility of our subsequent bioinformatic analyses and functional inferences regarding Cd detoxification and flavonoid accumulation in coix. These genes respond to Cd stress and play crucial roles in Cd uptake, efflux, sequestration, and antioxidation, thereby regulating Cd content in the roots, stems, leaves, and grains of coix.

## 4 Discussion

Plants exhibit toxicity symptoms, such as growth inhibition, under Cd stress, which might lead to yield reduction or even death. Though coix is a vital medicinal and edible plant, there have been very few reports addressing the responses of coix to heavy metal stress.

Plant height and tiller number are the most significant indexes for assessing plant resistance to Cd toxicity. In the current study, under Cd stress, plant growth was severely suppressed, which corroborated the results of previous studies on cotton (Daud et al., 2015), sorghum (*Sorghum bicolor*) (Zhan et al., 2017), wheat (Guo et al., 2019), and *Pleurotus* (Li et al., 2018). Furthermore, after treatment with 30 mg kg^−1^ Cd, the low Cd concentrations were transported and accumulated in coix, reaching 32.1, 14.8, 6.9, and 0.8 mg kg^−1^ in roots, stems, leaves, and grains, respectively. This finding indicated that coix possessed a weak Cd accumulation ability, which can aid the breeding of low-Cd coix cultivars.

In recent years, transcriptome sequencing has been increasingly applied to investigate gene response to abiotic stresses (Kashif et al., 2020; Chai et al., 2020; Huang et al., 2019b; Duarte et al., 2019). The molecular mechanisms underlying coix response to Cd stress remain unknown. Hence, the present study employed comparative transcriptome analysis of the control and Cd-treated coix. The root, stem, leaf, and grain samples were chosen for RNA-seq after 111 d of exposure to either 0 or 30 mg kg^–1^ CdCl_2_. For the coix transcriptome, 6.20–8.68, 7.63–8.34, 7.84–9.27, 7.22–8.28, 8.41–9.68, 6.84–7.66, 6.54–7.29, and 6.33–8.41 billion bases of clean reads were generated from CK-G, C-G, CK-J, C-J, CK-Y, C-Y, CK-Z, and C-Z, respectively. Additionally, expression levels of 12 randomly chosen DEGs were validated by qRT-PCR, whose expression patterns were found to be consistent with RNA-seq data (**Figure 9**). This finding indicated that the transcriptome sequencing of the coix was accurate and reliable for further analyses. In total, 1144, 2924, 3818, and 1702 DEGs, with 682, 942, 1907, and 877 upregulated and 462, 1982, 1911, and 825 downregulated genes, were identified under Cd stress in coix root, stem, leaf, and grain, respectively. Our results demonstrated that most DEGs were engaged in catalytic activity and binding in MF, cell and cell part in CC, and cellular process and metabolic process in BP.

Additionally, the KEGG pathway annotation analysis suggested that DEGs were primarily engaged in environmental adaptation, translation (root, stem, and leaf) or replication and repair (grain), transport and catabolism, signal transduction, and carbohydrate metabolism in roots, stems, leaves, and grains. GO and KEGG analyses demonstrated that most of the identified DEGs played key roles in the response of coix to Cd stress.

Plants normally use normal transporters for the uptake and root-to-shoot transport of Cd (Wu et al., 2019). Excessive Cd levels prevent the intake and transportation of Zn^2+^, Fe^2+^, Mn^2+^, etc., and the absorption of nutrients. In present study, Cd accumulation was significantly reduced in the order of roots, stems, leaves and grains, and it increased significantly with increasing Cd concentration in the soil. Similar results have been reported by previous studies on wheat (Guo et al., 2019), mung bean (Nahar et al., 2016), cotton (Daud et al., 2015), and poplar (Kieffer et al., 2009).

Expression analysis of transporter genes is essential to elucidate the mechanisms underlying Cd transportation and tolerance. The present study identified many transporter families, such as ABC, ZIPs, CAX, YSL, and MTP, to be engaged in heavy metal absorption, transportation, distribution, and tolerance (Zhao et al., 2001; Xie et al., 2015; Tsednee et al., 2014).

ABC transporters is the biggest transporter protein family. These transporters play a vital role in various metabolic processes, such as Cd tolerance, fatty acid import, hormone transport, osmotic homeostasis, and nutrient uptake (Park et al., 2012). ABC transporters have previously been shown to play essential roles in the absorption, transportation, sequestration, and detoxification of Cd in wheat (Wang et al., 2017). Cd induces the expression of ABC transporter ABCC3 in Arabidopsis and improves Cd tolerance mediated by phytochelatin (Brunetti et al., 2015). The present study found three differentially regulated ABC transporters in the root, including ABCG25, ABCG28, and ABCC3, all upregulated under Cd stress (**Supplementary Tables S18 and S19**). Thirteen ABC transporter genes were found to be differentially regulated in stems (**Supplementary Tables S20, S21**). Among them, five genes, ABCG11, ABCG31, ABCG8, ABCB19, and ABCB2, were significantly upregulated, while eight genes, ABCC10, ABCG36, ABCB11, ABCC4, ABCG43, ABCG50, ABCG48, and ABCC3 were significantly downregulated under Cd stress. Eight ABC transporter genes were found to be differentially regulated in the leaf (**Supplementary Tables S22, S23**). Among them, four genes, ABCB11, ABCC13, ABCB28, and ABCA7, were significantly upregulated, while four genes, ABCG16, ABCC9, ABCC10, and ABCB19, were significantly downregulated under Cd stress. Finally, eight ABC transporter genes were found to be differentially expressed in the grain (**Supplementary Tables S24, S25**). Among them, three genes, ABCB11, ABCB28, and ABCF5 were significantly upregulated, while five genes, ABCC4, ABCG43, ABCF2, ABCG50, and ABCG48, were significantly downregulated under Cd stress.

In this study, ZIPs, MTP, YSL, and CAX family members were also affected, but only four of these transporters were characterized as DEGs in the stem (**Supplementary Tables S20, S21**). Among them, one gene, MTP4, was significantly upregulated, while three genes, ZIP8, CAX2, and MTP5, were significantly downregulated under Cd stress. Four of these transporters were identified as DEGs in the leaf (**Supplementary Tables S22, S23**). Of them, three genes, IRT1, YSL13, and YSL12, were significantly upregulated, while one gene, CAX1a, was significantly downregulated under Cd stress. In several species, ZIPs play essential roles in the transportation of Zn, Cd, and Fe across the cell membrane. However, NRAMP transporters participate in metal absorption as well as homeostasis and are generally not significantly selective for divalent metal cations. Treating *Brassica rapa* and black nightshade (*Solanum nigrum* L.) with Zn or Cd increased the expression of NRAMP transporters, indicating that these transporters are linked to the accumulation and tolerance of heavy metals (Song et al., 2014). Additionally, many proteins related to heavy metal transportation/detoxification are found in coix under Cd stress. These proteins possess a heavy-metal-associated domain (HMAD) and are potentially implicated in toxic metal transportation and toleration (Rono et al., 2019). For example, RNA-seq showed that *HIPP45* was downregulated under Cd stress.

It has been widely reported that TFs are engaged in regulating stress responses. TFs, like ethylene-responsive (ERF), basic leucine zipper (bZIP), GRAS domain family (GRAS), myeloblastosis protein (MYB), and WRKY, regulate gene expression under Cd stress (Chen et al., 2020). Many WRKY genes were activated by heavy metal stress in *Arabidopsis* (Liu et al., 2009). In rice, many TFs, such as MYB, AP2, NAC, and WRKY, are significantly upregulated under Cd stress (He et al., 2015; Ogawa et al., 2009). In kenaf, MYB, bHLH, NAC, and ZIP TF families have been found to be differentially regulated under Cd stress (Chen et al., 2020). The expression of many TFs, such as MYB, HSF, AP2, and C2H2, is induced by Cd in *Arabidopsis* (Weber et al., 2006). In addition, in *Arabidopsis*, 176 proteins containing zinc-finger domains have been identified (Englbrecht et al., 2004), many of which play vital roles in stress responses and the development of plants. Furthermore, in *Arabidopsis*, ZAT6, a zinc-finger TF, positively regulates Cd tolerance via the glutathione-dependent pathway (Chen et al., 2016). In another study, MYB, GRAS, NAC TF-like, bHLH, bZIP, and ERF TFs were found to be differentially expressed in two different *Vicia sativa* varieties under Cd stress (Rui et al., 2018).

This study reveals a transcriptional regulatory pattern distinct from species such as *Arabidopsis* and rice: under Cd stress, most differentially expressed transcription factors like WRKY and MYB in coix are significantly upregulated only in the roots and grains, while their response in the stems and leaves is minimal. This unique tissue-specific pattern may be intrinsically linked to its known low Cd accumulation trait. We hypothesize that this reflects a “root-restricted” defense strategy in coix. Specifically, the root system activates extensive transcriptional reprogramming to efficiently initiate mechanisms such as Cd fixation, sequestration, and efflux, thereby minimizing Cd transport to the above-ground parts. The successful restriction of Cd accumulation in the aerial tissues results in weaker stress signaling in the stems and leaves, thus obviating the need for large-scale, energy-consuming transcriptional responses. Coix activates several TFs that regulate the expression of genes engaged in the alleviation of Cd toxicity. Further analyses might help decipher the regulatory network associated with Cd stress in coix.

## 5 Conclusion

This study provides the first multi-tissue transcriptomic atlas of coix under cadmium stress, revealing a novel root-restricted defense strategy that underlies its low cadmium accumulation phenotype. Our key mechanistic insights are threefold: (1) Concerted activation of the phenylpropanoid and flavonoid pathways across roots, stems, and leaves enhances lignification for cadmium immobilization and non-enzymatic antioxidant defense. (2) Above-ground tissues (stems and leaves) further deploy the glutathione pathway for antioxidant defense, enzymatic detoxification and chelation. (3) This multi-layered, tissue-coordinated mechanism effectively limits cadmium translocation to the grains. These findings provide a genetic foundation for breeding low-cadmium coix varieties and advance our understanding of cadmium tolerance mechanisms in medicinal and edible plants.

## Data Availability

The datasets presented in this article are not readily available because the raw data was not stored. The third party company have compiled and provided five sets of results which have been published as **Supplementary Materials**.
