# Supplementary material for: Transcriptome analysis revealed key genes and pathways related to cadmium tolerance and accumulation in coix (Coix lacryma-jobi L.)
Source: Front Plant Sci. 2026 Feb 27;16:1660959. doi: 10.3389/fpls.2025.1660959 (PMC12985850; doi:10.3389/fpls.2025.1660959)
Supplement: Supplementary file 1 [file DataSheet1.zip › Supplemenatry Tables/Table S1.docx]

**Table S1.** The primer sequences for DEGs qRT-PCR validation.

| Unigene ID | Name | Primer sequences（Sense） | Primer sequences（Anti-sense） |
| --- | --- | --- | --- |
| Unigene0048533 | *ABCG28* | 5' TATCTTCCTGGGGGAAGGGG 3' | 5' TTGCTTGTTCTCAGCGGGAT 3' |
| Unigene0101091 | *ABCC3* | 5' TCACGATAGCGCACAGGATC 3' | 5' AAGCAACAACCAACAGGGGA 3' |
| Unigene0004542 | *MTP4* | 5' AGCTCCTCCGGAAATGCTTC 3' | 5' AAGTCCTCTGGGAGCTCGAT 3' |
| Unigene0090192 | *IRT1* | 5' GTGTACAGGGAGAACAGCCC 3' | 5' AGGAAGGAGAGGAGCTGGAG 3' |
| Unigene0092438 | *ABCA7* | 5' TGCTGTCGTCTGTGATGACC 3' | 5' TCCCAGCTCCATTAGGACCA 3' |
| Unigene0099561 | *YSL13* | 5' GTCGAGGTTCATCCCGATCC 3' | 5' GGCGAATGCGTCTGCTTTAG 3' |
| Unigene0099564 | *YSL12* | 5' ATGTGGCCTCTCATTCGCAA 3' | 5' GTTGTACAGGCCGTCTCCAA 3' |
| Unigene0006961 | *ABCB11* | 5' GGAAGCAAGGGGAAGCATCT 3' | 5' CGCCTGACAGTTGGATTCCT 3' |
| Unigene0093309 | *HCT4* | 5' ACTACAGCGGCTTCGTGTAC 3' | 5' GGCGAACACCTTCCTGAACT 3' |
| Unigene0035743 | *CYP93G1* | 5' GCACTTCCAGTACATGCCCT 3' | 5' GTCGATCTGCTTGTTGGTCG 3' |
| Unigene0056120 | *CYP75B3* | 5' ACTTCGAGCTCATCCCGTTC 3' | 5' CGTATGCCTCCTCCATGTCC 3' |
| Unigene0090121 | *CYP73A12* | 5' GCCGACTTGGTCTTCACTGA 3' | 5' TCCATCTCGGTCTCCCACAT 3' |
| Actin | *Actin* | 5' GCTACGAGATGCCTGATG 3' | 5' CCACTGAGGACAACATTACC 3' |
