# Supplementary figures and images for: Transcriptome analysis revealed key genes and pathways related to cadmium tolerance and accumulation in coix (Coix lacryma-jobi L.)
Source: Front Plant Sci. 2026 Feb 27;16:1660959. doi: 10.3389/fpls.2025.1660959 (PMC12985850; doi:10.3389/fpls.2025.1660959)

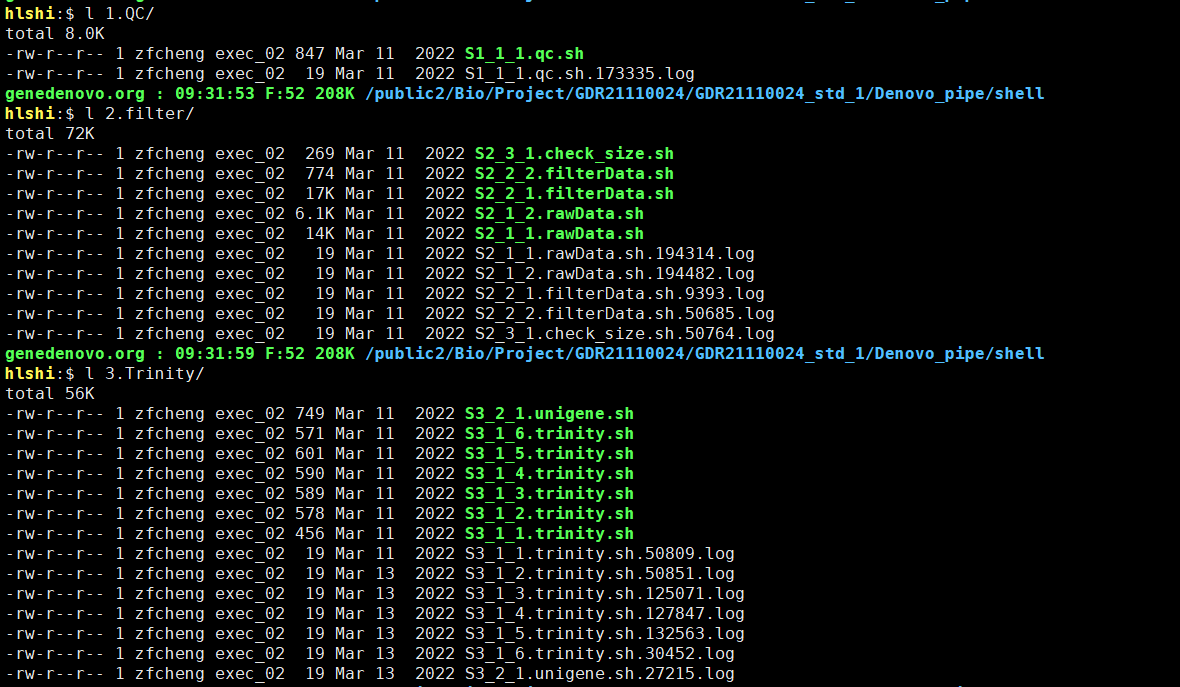

Supplement: Supplementary file 4 [file Supplementaryfile3.zip › 3.pipe_log/1-3.png]

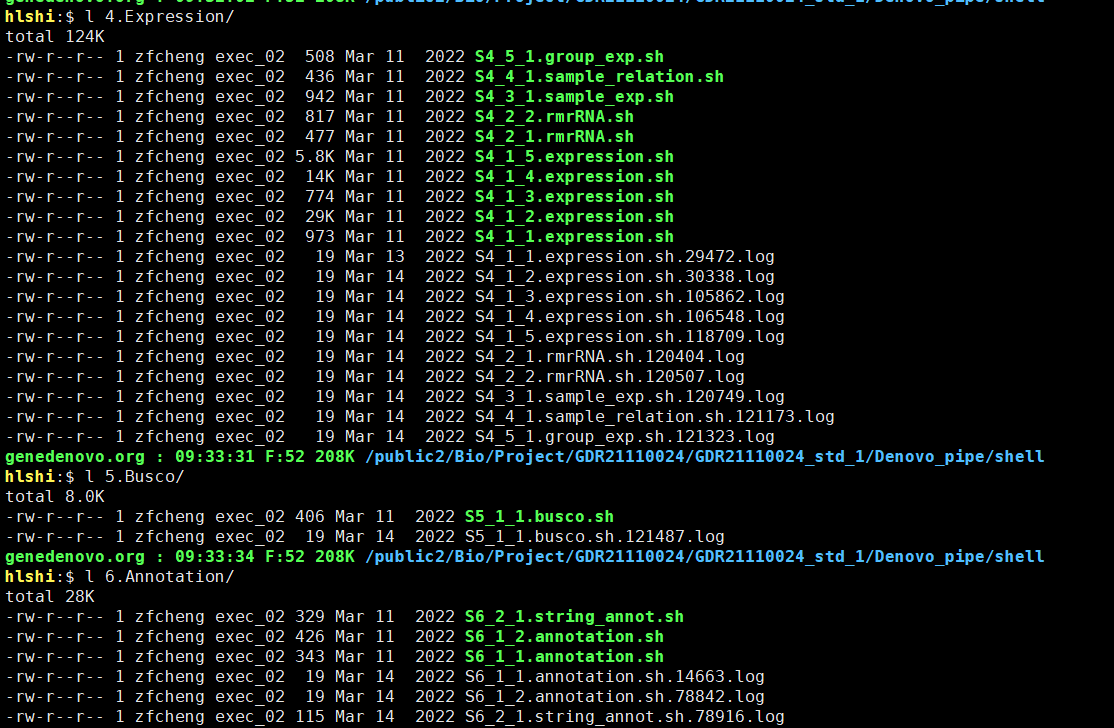

Supplement: Supplementary file 4 [file Supplementaryfile3.zip › 3.pipe_log/4-6.png]

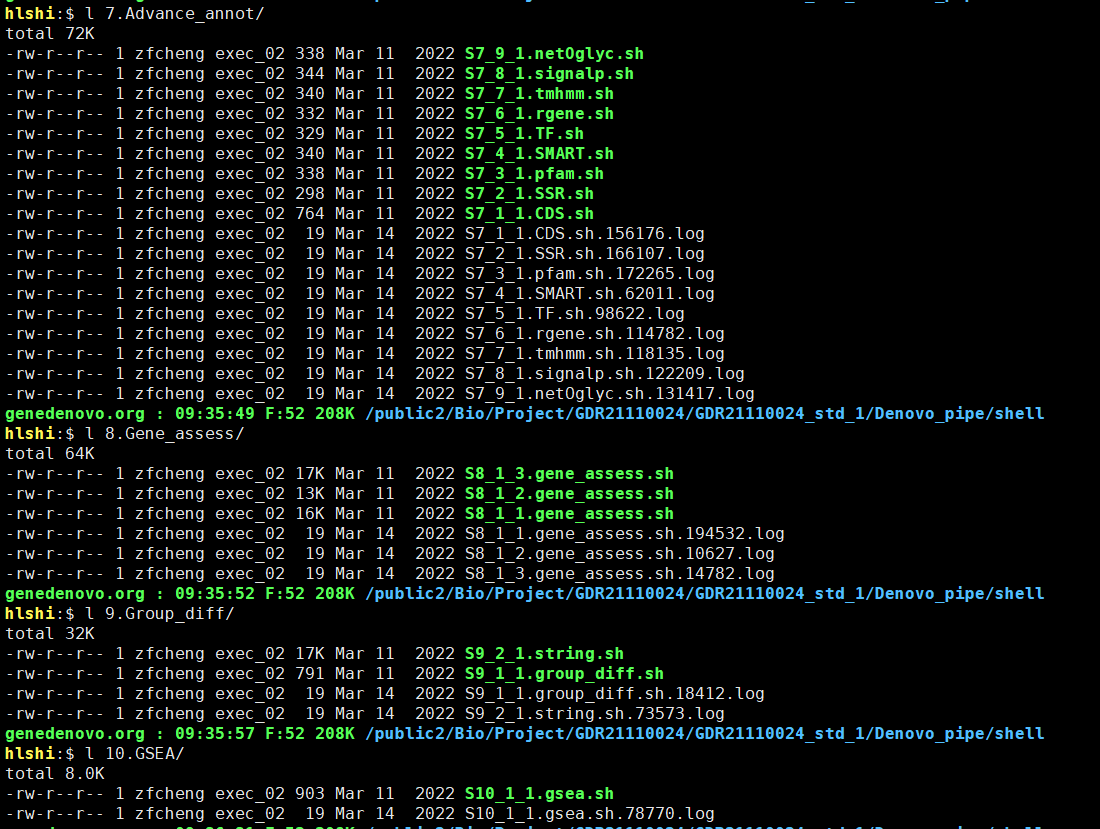

Supplement: Supplementary file 4 [file Supplementaryfile3.zip › 3.pipe_log/7-10.png]
